# Delivery of Antioxidant and Anti-inflammatory Agents for Tissue Engineered Vascular Grafts

**DOI:** 10.3389/fphar.2017.00659

**Published:** 2017-09-21

**Authors:** Kenyatta S. Washington, Chris A. Bashur

**Affiliations:** Department of Biomedical Engineering, Florida Institute of Technology, Melbourne FL, United States

**Keywords:** drug delivery, vascular tissue engineering, inflammation, gasotransmitters, antioxidants, carbon monoxide releasing materials

## Abstract

The treatment of patients with severe coronary and peripheral artery disease represents a significant clinical need, especially for those patients that require a bypass graft and do not have viable veins for autologous grafting. Tissue engineering is being investigated to generate an alternative graft. While tissue engineering requires surgical intervention, the release of pharmacological agents is also an important part of many tissue engineering strategies. Delivery of these agents offers the potential to overcome the major concerns for graft patency and viability. These concerns are related to an extended inflammatory response and its impact on vascular cells such as endothelial cells. This review discusses the drugs that have been released from vascular tissue engineering scaffolds and some of the non-traditional ways that the drugs are presented to the cells. The impact of antioxidant compounds and gasotransmitters, such as nitric oxide and carbon monoxide, are discussed in detail. The application of tissue engineering and drug delivery principles to biodegradable stents is also briefly discussed. Overall, there are scaffold-based drug delivery techniques that have shown promise for vascular tissue engineering, but much of this work is in the early stages and there are still opportunities to incorporate additional drugs to modulate the inflammatory process.

## Introduction

Cardiovascular disease is the leading cause of death worldwide, and consists of several different conditions (Lloyd-Jones et al., 2010; Benjamin et al., 2017). The etiology of many of these conditions, including coronary and peripheral artery disease, is the accumulation and maturation of atherosclerotic plaques within the arteries. These plaques can constrict the arteries, and plaques vulnerable to erosion or rupture can cause emboli (Bentzon et al., 2014). Treatment options depend on the severity of the occlusion. For patients requiring intervention, current options include balloon angioplasty, stent grafts, and autologous bypass grafts. In addition, tissue engineering is being investigated as an alternative to produce vascular grafts, especially for the greater than 30% of patients exhibiting multiple plaques that do not have viable veins for grafting (Curi et al., 2002; Albers et al., 2003; Klinkert et al., 2004). Another significant target for tissue engineered vascular grafts (TEVGs) is pediatric patients who need a graft that can grow with them. However, each of these intervention options have their limitations and require consideration of strategies to control the inflammatory response and inflammation-related complications. For example, restenosis is an important concern for stent grafts and thrombosis and intimal hyperplasia are major concerns for tissue engineered grafts (Shinoka et al., 1998; Kereiakes et al., 2016). Strategies to modulate the inflammatory response include the properties of the grafted material as well as traditional pharmacological approaches (Nguyen et al., 2013). This review focuses on drug delivery approaches used to distribute specific antioxidant or anti-inflammatory agents within TEVGs. The application of tissue engineering and drug delivery concepts to stent grafts is also briefly discussed. For this review, we focus only on treatment options for small-diameter vessels (i.e., <6 mm) such as the coronary and peripheral arteries.

## Vascular Bypass Graft Options

Nearly one in every five deaths in the United States is due to coronary heart disease. In addition, occlusion of small-diameter peripheral arteries in the legs often leads to amputation (Curi et al., 2002; Albers et al., 2003; Klinkert et al., 2004). Combined, these diseases create a significant clinical need for treatment options. The gold standard for bypass grafting in patients with blocked arteries is autologous vessels (Klinkert et al., 2004; Chew et al., 2005). Saphenous veins work quite well, but are unavailable in more than 30% of affected patients because of systemic vascular disease (Klinkert et al., 2004). The internal mammary artery is also an option, but it is not typically long enough for bypass in peripheral arteries. Non-degradable, synthetic vascular prostheses, such as expanded poly(tetrafluoroethylene) (ePTFE) have been used, but these prostheses tend to promote inflammatory responses that lead to thrombosis, intimal hyperplasia, and a high failure rate (Klinkert et al., 2004; Chlupác, 2009). Stent grafts also have the concern with exaggerated intimal hyperplasia, with bare metal stents leading to in-stent restenosis in 15 to 60% of patients within 12–24 months (Dussaillant et al., 1995; Waksman et al., 2000; Chen et al., 2006). Tissue engineering approaches have the potential to produce alternative self-repairing grafts that remodel and integrate with the surrounding artery.

## Tissue Engineered Grafts

Tissue engineering involves applying the knowledge of how tissues grow and how cells interact with their surroundings to generate a graft (MacArthur and Oreffo, 2005). These approaches involve a carefully designed scaffold that can be degraded as cells (i.e., the living component) deposit and organize new tissue. The cells are either pre-seeded outside the body or are recruited *in vivo* after implantation of an acellular graft. Either way, the host response after implantation in the body will determine whether the graft will remain viable. Extended inflammatory responses are known to prevent the development of a functional endothelial layer in the lumen of the vessel. This has been demonstrated extensively in native vessels with atherosclerosis (Lerman and Zeiher, 2005) and in synthetic ePTFE grafts (Clowes et al., 1986). This endothelium is one of the important parameters needed to prevent graft occlusion (Shojaee and Bashur, 2017). Further, inflammatory products such as oxidized low density lipoprotein (ox-LDL) have been linked to both endothelial cell and smooth muscle cell (SMC) dysfunction. The Rosenbaum group has demonstrated that hypercholesterolemia in C57B1/6 mice prevents endothelial cell healing through an increase in oxidative stress (e.g., ox-LDL), and they hypothesized that the multiple sources of oxidative stress present in the clinical setting may be a challenge for treating cardiovascular disease (Rosenbaum et al., 2012). For SMCs, ox-LDL has been shown to bind to lectin-type oxidized LDL receptor-1 (LOX-1), activating the nuclear factor-kappa beta (NF-κβ) transcription factor, and leading SMCs to switch to an activated phenotype that has a role in intimal hyperplasia and stenosis (Draude et al., 1999; Orr et al., 2010). These results of extended inflammation and oxidative stress are also important considerations for tissue engineered vascular grafts.

A range of scaffold properties, such as composition, topography, and mechanical compliance, are important for controlling the inflammatory response, tissue generation, and general graft viability (Bashur et al., 2012). These impacts are reviewed in detail in other review articles (Drury and Mooney, 2003; Cheung and Lu, 2007). Importantly, tissue engineering scaffolds can also serve as drug delivery systems to provide local and controlled release of pharmacological agents to the tissues of interest. The release of bioactive molecules from the scaffold is a technique that has been used to try to reduce the intimal hyperplasia and stenosis, and improve the long-term viability of vascular grafts.

## Drugs Released for Vascular Tissue Engineering

A wide variety of small-molecule drugs, growth factors, and other bioactive molecules have been released from tissue engineered scaffolds. These drugs are often added to either promote aspects of tissue growth or modulate the inflammatory response, with many having dual roles (Boehler et al., 2011). However, relatively few drugs have been delivered for the generation of vascular grafts for artery replacement. This is especially noticeable when comparing with approaches to engineering microvasculature such as capillaries, which primarily involves pharmacological approaches such as vascular endothelial growth factor (VEGF) delivery (Lee et al., 2011). **Table 1** lists bioactive molecules that have been released from vascular scaffolds. Most of these molecules are antioxidant or anti-inflammatory compounds, and often they are presented in a non-traditional method through integration with the scaffold. These categories of drugs and their pharmacodynamics are discussed in detail in later sections. Degradation products of natural and synthetic macromolecules contained within a scaffold also often have pro- or anti-inflammatory properties (Hance et al., 2002; Higgins et al., 2003). This is discussed in detail elsewhere, but will not be discussed in this review (Badylak, 2007; Malafaya et al., 2007).

**Table 1:** Bioactive molecules released from vascular scaffolds.

Molecule	Response	Reference	
**Antioxidants**			
Ascorbic acid/Citric acid	Maintained cellular viability in high ROS environment	Gregory et al., 2011; van Lith et al., 2014	
Penta-galloyl glucose	Reduced degradation of the scaffold by matrix metalloproteases	Chuang et al., 2009; Kostyuk et al., 2011; Simionescu et al., 2011; Chow et al., 2013	
**Gasotransmitters**			
Carbon monoxide	Inhibiting the expression of pro-inflammatory cytokines; promoting interaction with local cell target	Otterbein et al., 2000; Bohlender et al., 2014; Michael et al., 2016	
Nitric oxide	Mediates vasodilation and inhibit platelet aggregation	Gewaltig and Kojda, 2002; Frost et al., 2005; Gregory et al., 2011; Liang et al., 2015	
*S*-nitrosothiols	Induced vasodilation	Gregory et al., 2011; Kumari et al., 2014; Liang et al., 2015
**Glycosaminoglycans**		
Heparin	Promoted endothelialization and SMC proliferation	Greisler et al., 1992; Shafiq et al., 2016; Spadaccio et al., 2016
**Growth factors and other Proteins**		
Fibroblast growth factor-1	Anticoagulant; vessel sprouting mediates interaction with ECM	Greisler et al., 1992; Gosselin et al., 1996; Zhang and Suggs, 2007
Anti-CD34 antibody	Increased endothelialization	Bhattacharya et al., 2000; Ravi and Chaikof, 2010
Transforming growth factor beta 1 (TGF-β1)	Promoted contractile protein expression by SMCs; reduced ring thickness; and promoted TEVG remodeling	Strobel et al., 2017

It is important to note that anti-inflammatory drugs such as paclitaxel, which are commonly used for drug-eluting stents (Burt and Hunter, 2006), are not typically useful for tissue engineering approaches because they also prevent cell proliferation and tissue deposition (Sollott et al., 1995; Axel et al., 1997; Farb et al., 2001). Tissue deposition is necessary in the early stages of tissue engineered graft generation, but at later time points the new tissue deposition should decrease and the cells should become more similar to those in the native environment. This is the reason why most tissue engineering research focuses on strategies that modulate and quickly resolve, but not avoid, the inflammatory response. The importance of this modulation is demonstrated by Roh et al. (2010) with their bone marrow mononuclear cell (BMC) pre-seeded TEVG. Specifically, they demonstrated that the success of their graft in a mouse model is due to an inflammation-mediated process where the BMCs released large amounts of monocyte chemoattractant protein-1 (MCP-1), leading to rapid monocyte recruitment. There are some strategies that have added the antibiotic doxycycline to reduce matrix metaloproteinase expression and preserve matrix structure (Venkataraman et al., 2014). However, doxycycline has also been shown to inhibit endothelial cell proliferation, which will prevent the formation of a functional endothelium (Bendeck et al., 2002). In this case, careful control over the dose and timing of the anti-inflammatory agent would be needed if it were incorporated within a tissue engineering strategy.

## Drug Delivery From Scaffolds

Delivery of bioactive compounds from tissue engineered scaffolds has several benefits. It provides more local delivery of the compound of interest to impact the cells attached to the scaffold and those at the surrounding anastomosis as well as reduce the overall drug loading required. This can be especially important for peptide or oligosaccharide therapeutics that have limited stability with systemic circulation (Yu et al., 2009). It is also important to note that the engineered grafts are implanted as part of the surgery, so additional surgical procedures or injections are not required for controlled drug delivery. Finally, tissue engineered scaffolds allow for several ways to incorporate pharmaceutical agents and control their release rate to deliver a dose within the therapeutic window at the time desired for specific applications.

**FIGURE 1:**
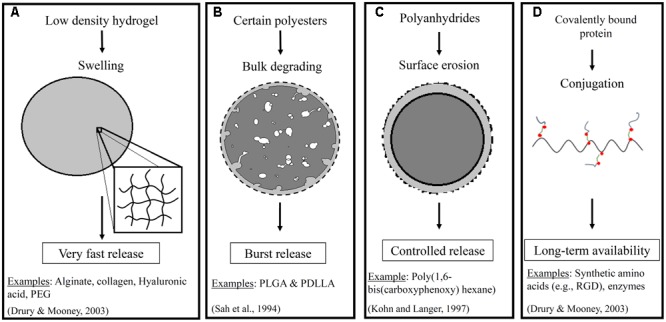
Illustrates a few important properties of scaffolds that impact drug release. These include **(A)** swelling of low density hydrogels, **(B)** bulk degrading polyesters, **(C)** surface erosion in polymers such as polyanhydrides in an aqueous environment, and **(D)** covalent conjugation of proteins. The resulting impact on the release profiles including very fast, burst, controlled, and extended or long term availability, respectively, for a representative pharmaceutical agent. The dashed line represents the initial boundary of the material (Sah et al., 1994; Drury and Mooney, 2003; Ratner et al., 2004).

The way that a drug is incorporated within the scaffold, and the composition of the scaffold itself, have important impacts on the success of a delivery strategy. **Figure 1** shows an illustration of some of the important properties of the scaffold and the impact on the release profile for a representative drug (i.e., a growth factor). Briefly, a range of material-types have been used in the scaffold for vascular tissue engineering (Lee and Shin, 2007). These include naturally derived materials (e.g., collagen, fibrin, and hyaluronan) that are added to better mimic the native environment found in tissue regeneration and wound healing. They also include synthetic materials such as poly(ethylene glycol), polyesters, and polyurethanes that can provide a range of physical and chemical properties to the system. The polyesters include commonly used poly(α-hydroxy esters) – e.g., poly(𝜀-caprolactone) (PCL) and poly(glycolic acid) (PGA) (Greisler, 1982) – as well as poly(glycerol sebacate) (PGS) (Wu et al., 2012). The polyurethanes that have been used for TEVGs are segmented elastomers (e.g., polyester urethane ureas) (Soletti et al., 2011). One of the reasons why the choice of material is important is that it impacts the release of pharmaceutical agents to the cells. For example, many polyesters provide a quick burst release of the drug that can be helpful in situations such as delivery of a growth factor that acts at the early stages of inflammation (Chen et al., 2010). This release profile is the quickest for the non-crystalline polyesters such as poly (lactic-co-glycolic acid) (PLGA) and poly (D,L-lactic acid) (PDLLA) (Sah et al., 1994). However, a more extended release profile is often desirable, and polymers such as poly(hydroxyalkanoates) are better able to provide this profile. One example of a poly(hydroxyalkanoate) used in tissue engineered scaffolds is poly(3-hydroxybutyrate) (PHB). PHB has been shown to exhibit surface erosion that can lead to sustained release (Ulery et al., 2011). Co-polymerization of PHB with other polymers has even been performed in some studies to speed up the release profile. The similar molecule poly(4-hydroxybutyrate) had been used in vascular grafts both within the fibers and as a coating, but it was not used to release any traditional pharmacological compounds (Opitz et al., 2004; Hoerstrup et al., 2006). Finally, it is important to note that preferred scaffold materials for release of either hydrophobic or hydrophilic drugs will vary.

The way that a scaffold is processed into a three-dimensional form for grafting will also impact the release profile. There are several different types of methods that are used to produce vascular tissue engineered scaffolds, such as electrospinning, freeze-drying, and self-assembly (Drury and Mooney, 2003; Buttafoco et al., 2006; Norotte et al., 2009; Birthare et al., 2016; Michael et al., 2016). Many of these methods aim to generate nanometer or micrometer sized fibers that form a three-dimensional tubular scaffold. These different methods can impact the pharmacology for several reasons, including the stability of the drug. Differences in surface area exposed to fluids will also impact the release rate. Finally, more advanced methods can be used for unique timing for release of the drug. For example, a lot of work is currently being performed with materials that will release a drug in response to an applied stimuli (e.g., temperature, pH, or light) (Schmaljohann, 2006). In addition, sometimes the scaffold is designed for sequential release of drugs when needed. For example, Jaklenec et al. (2008) developed a microparticle system that can sequentially release insulin-like growth factor-1 (IGF-1) and then transforming growth factor β_1_ (TGF- β_1_). The ultimate goal of this system is described as improving engineered cartilage tissue, but only cell lines (i.e., MCF-7 cells and HT-2 cells) were used in this particular study (Jaklenec et al., 2008). In addition, the Mooney group has developed a microparticle system that can release angiogenic factors sequentially [i.e., VEGF and then platelet derived growth factor (PDGF)] (Richardson et al., 2001). They have also shown that delaying release of the maturation factor PDGF allowed for increased vascular remodeling in Lewis rats and non-obese diabetic (NOD) mice (Brudno et al., 2013). However, these multi-step delivery strategies have not yet been employed with TEVGs. An interesting approach that has been used for TEVG applications is incorporation of gelatin microspheres within vascular tissue rings to deliver TGF-β_1_ to SMCs within the ring (Strobel et al., 2017). This is one of the few studies that have delivered drugs to promote the remodeling of tissue engineered vascular grafts in particular. The Rolle group found that TGF-β_1_ treatment promoted contractile protein expression by the SMCs and resulted in reduced ring thickness (Strobel et al., 2017). In the following sections, these drug delivery concepts will be included within specific vascular tissue engineering examples where modulation of the inflammatory response was attempted.

## Modulating Vascular Graft Inflammation

In a tissue engineering strategy, it is important to consider both inflammation present in diseased tissues and the additional inflammatory stimuli presented by TEVGs. The drug delivery strategy can involve modulating white blood cell recruitment as well as later steps in the inflammatory process. The control over macrophage phenotype especially has become an area of significant research in the tissue engineering field. The spectrum of macrophage phenotypes from traditionally activated pro-inflammatory M1 to tissue depositing M2a or anti-inflammatory M2b macrophages has been described extensively in recent literature (Mosser and Edwards, 2008; Sridharan et al., 2015). This offers an important target for drug delivery, with the timing being very important. M2 macrophages have been described as both positive “pro-healing” or negative “pro-fibrotic,” and they can behave both ways (Keophiphath et al., 2009; Spiller et al., 2014). For tissue engineering, some degree of tissue deposition is needed for healing, but both the release of inflammatory cytokines and tissue deposition must be quickly resolved. Otherwise, tissue encapsulation and foreign body giant cell formation can occur (Anderson, 1988). Overall, the goal is that the inflammatory response initiates remodeling but subsides quickly to allow the presence of vascular cells exhibiting a phenotype similar to those in a healthy vessel as well as extracellular matrix maturation.

The diseased cells and tissue present in the native artery surrounding the graft are also important for the inflammatory response in the graft. For this reason, an understanding of the native tissues is needed. Different types of cells are found within the specific layers of arteries (Sell et al., 2009) and these cells play an important role in both healthy and pathological tissue. The confluent endothelial cells in the intimal layer prevent initiation of the coagulation cascade, and provide paracrine signals to SMCs within the medial layer that help to maintain the quiescent SMC phenotype. However, endothelial cells in diseased tissue do not exhibit proper function (e.g., loss of cell-cell signaling though gap junctions, in particular connexins 37, 40, and 43) (Liao et al., 2001; Verma et al., 2003) and this can contribute to SMC hyper-proliferation. SMCs are found in several layers in the middle of the vessel and are important for vascular tone. However, SMCs activation and migration to the intima is one of the main factors for atherosclerotic lesion formation and intimal hyperplasia (Inoue et al., 1998). Finally, the adventitial layer is made of fibroblast and also pericytes with stem-cell like characteristics. All of these vascular cell-types from the surrounding artery, when diseased, are able to release pro-inflammatory cytokines [e.g., interferon-gamma (IFN-γ) and MCP-1] that can impact the graft (Doran et al., 2008). They are also able to migrate into the graft from the anastomosis. Overall, these biological processes offer many pharmacological targets to improve tissue engineered vascular graft viability (Naito et al., 2011). Many of the pharmacological and non-pharmacological strategies that have been used to generate TEVGs modulate steps in the inflammatory process, even if that is not the stated intention of the study.

## Pharmacology Examples for Vascular Grafts

The few pharmacological agents delivered from vascular tissue engineering scaffolds for modulating inflammation have been listed previously in **Table 1**. The following sections provide details for two of these types of drugs: general antioxidant compounds and gasotransmitters.

### Antioxidant Compounds

Antioxidant compounds are often delivered to counter the effects of free radicals – i.e., reactive oxygen species (ROS) and reactive nitrogen species (RNS) – that are found in the blood vessels. The free radicals are produced in the body either by normal cell metabolism or in response to external sources including radiation, cigarette smoking, and pollution. Further, free radicals can either be beneficial or toxic, and the specific concentration levels are important. ROS and RNS can demonstrate positive benefits on cellular responses (e.g., redox signaling and cell survival) and immune function at low to moderate levels (Sena and Chandel, 2012; Yan, 2014). At higher concentrations, they display oxidative stress that can be harmful to cells (Pham-Huy et al., 2008). These high concentrations are what TEVGs will be exposed to when grafted into diseased arteries.

There are a diverse range of antioxidant compounds that have been tested to determine their ability to delay atherosclerotic plaque generation in animal models. These include studies showing that probucol reduces the fatty-streak lesions in LDL receptor deficient rabbits (Carew et al., 1987), that dihydrotanshinone I reduced atherosclerotic plaque formation and shrunk the necrotic core in apolipoprotein E-deficient mice (Zhao et al., 2016), and that supplementation of dietary antioxidants (i.e., a mixture of vitamin E, vitamin C, selenium, zinc, copper, manganese, *N*-acetylcysteine, and glutamine) reduced atherosclerotic plaque formation in hypercholesterolemic rabbits (Leborgne et al., 2005). Other plant polyphenols have also been tested for responses relevant for vascular grafts (Fusco et al., 2007). For example, Kostyuk et al. (2011) analyzed antioxidant properties of several plant polyphenols and their effects on the signal response of human umbilical vein endothelial cells (HUVECs) to oxLDL and lipopolysaccharides (LPS) *in vitro*. These plant phenols include resveratrol that can be found in red wine and has been shown to have antioxidant effects (Gülçin, 2010). The Kostyuk study concluded that resveratrol partially reduced expression of genes associated with oxidative stress and inflammation [e.g., MCP-1, interleukin 8 (IL-8), and inducible cyclooxygenase 2 (COX-2)], although the biological mechanism for the results needs to be further investigated. However, the American Heart Association mentions that additional research (e.g., randomized clinical trials) is needed to verify the benefit of supplementary doses of antioxidants. One potential concern for efficacy in the clinical environment is the low bioavailability of many of the antioxidant compounds when delivered systemically due to their high reactivity (Goszcz et al., 2015). The incorporation of antioxidant compounds within implanted tissue engineered grafts may be able to retain the antioxidant activity and overcome some of the limitations with systematic delivery.

Few of these antioxidant compounds that have demonstrated promise in animal models for atherosclerosis have been tested for vascular tissue engineering. The polyphenol penta-galloyl glucose (PGG) has been incorporated within tissue engineered vascular grafts (Chuang et al., 2009). This polyphenol is a component of tannic acid that can be extracted from multiple plants and has been suggested to have antioxidant properties (Georgé et al., 2005). However, it is the collagen and elastin binding properties that are the main reason why Simionescu et al. (2011) have incorporated it within naturally derived elastin-based tubular scaffolds. PGG was incorporated by binding to elastin-based scaffolds and was shown to reduce degradation of the scaffold by matrix metalloproteases, with MMP-2 and MMP-9 specifically identified (Chuang et al., 2009). Scaffold treatment with solution concentrations up to 0.3% PGG provided maximum scaffold stability and did not exhibit signs of cytotoxicity. In more recent studies, the Simionescu group has also considered the suggested antioxidant properties (Chow et al., 2013) of PGG in their elastin-based scaffolds. This strategy is still under investigation, and more information will be needed such as details of the PGG release. Studies with PGG modified scaffolds have recently been performed in a rat model with direct arterial grafting (Pennel et al., 2014).

The incorporation of ascorbic acid (AA) and citric acid is another antioxidant strategy for vascular tissue engineering that has been tested within the arterial environment (van Lith et al., 2014). AA, or vitamin C, is a well-known free radical scavenger in the body. van Lith et al. (2014) also considered citric acid because it can stabilize AA and help to improve the antioxidant properties of the scaffold. In their strategy, they used a non-traditional approach to deliver the antioxidant to vascular cells that may attach to the scaffold. Instead of adsorbing or trapping the material within the scaffold and providing release for a limited time, they incorporated both AA and citric acid within a co-polymer (5:1 ratio of citric acid/AA). The extended time that these antioxidants are provided as part of the scaffold may be important considering the chronic inflammatory environment found within the surrounding diseased artery. They show that gradual release of AA over 2 months with degradation of the scaffold may also be important for the scaffold’s antioxidant properties (van Lith et al., 2014). *In vitro* studies demonstrated that HUVECs maintained their viability in a high ROS environment generated by exposing the cells to 50 μM menadione, unlike controls without the antioxidant polymer. They tested their antioxidant polymer as a coating within a traditional PTFE vascular graft in a guinea pig (van Lith et al., 2014). Their *in vivo* results were less pronounced (e.g., the effect on neo-intimal hyperplasia), but they are further investigating the mechanism for this. Finally, the antioxidant molecule that has been investigated most extensively for vascular tissue engineering is nitric oxide (NO). NO is discussed in detail in the gasotransmitter section below.

### Gasotransmitters

There are several inorganic gasses that have a unique role in cellular signaling in biological pathways, and these are called gasotransmitters. Examples of these type of molecules include NO and carbon monoxide (CO). Both of these molecules are naturally occurring vasodilators (Wu and Wang, 2005; Cirino et al., 2006). They also have been shown to exhibit anti-inflammatory effects at certain doses. For this reason, inhaled NO is approved for treating neonatal patients with pulmonary hypertension (Chotigeat et al., 2007; Major et al., 2010), and inhaled CO therapy has been investigated in pre-clinical models and is currently in a phase II clinical trial for treating pulmonary fibrosis in adult patients (Lowson, 2004). However, dose is critical for these gasotransmitters. Both of them have been shown to have anti-inflammatory effects at appropriate doses, but high doses provide the opposite pro-inflammatory effect (Otterbein et al., 2000; Gewaltig and Kojda, 2002; Motterlini et al., 2002). There are some similarities between the mechanisms for each of the molecules as described below, although there are also some important differences (Zhuo et al., 1993; Motterlini, 2007; Liang et al., 2015).

#### Mechanisms for Endogenous NO

**FIGURE 2:**
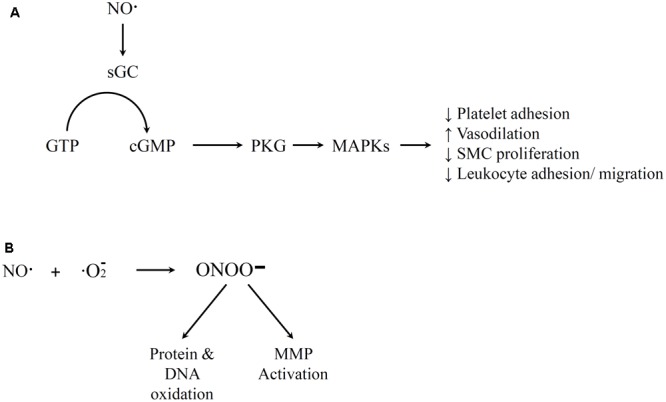
Illustration of nitric oxide (NO) Pathways. **(A)** Positive medical effects that involve guanylate cyclase activation by NO, conversion of guanosine triphosphate (GTP) to cGMP, and activation of downstream PKG that lead to various vascular homeostasis impacts. **(B)** Free radical reaction of NO and oxygen that yields peroxynitrite that leads to negative impacts of NO. References for this figure are listed in the text.

The free radical NO mediates a number of physiological functions. The Moncada group has described how NO production is one of the most important function of the endothelium (Moncada and Higgs, 1991). It regulates blood flow and cell–cell communication, and it is also a key component in the anti-atherosclerotic properties of the endothelium (Wever et al., 1998). NO is generated in the body when nitric oxide synthase (NOS) enzymes degrade L-arginine (Wever et al., 1998). Endothelial NOS is NADPH and Ca^2+^/calmodulin dependent. The biological impacts of NO have been extensively investigated, and are only briefly summarized below. A variety of stimuli such as bradykinin have been shown to initiate NO generation (Palmer et al., 1987; Böger et al., 1996). NO is a very reactive gas with a short half-life in the body. At healthy levels, NO often reacts with heme-containing molecules, either through the protein moity to form *S*-nitrosothiols or directly with the metal ion. For example, NO activates guanylate cyclase (GC), converts guanosine triphosphate (GTP) to cyclic guanosine monophosphate (cGMP), and activates protein kinase G (PKG) that can lead to several results important for vascular hemostasis (**Figure 2A**) (Ignarro et al., 1987). Healthy NO signaling has several impacts including mediating vasodilation, inhibit platelet aggregation, decreasing SMC proliferation, and reducing leukocyte adhesion and migration (Garg and Hassid, 1989; Jeremy et al., 1998; Sarkar and Webb, 1998; Gewaltig and Kojda, 2002). However, NO can also react with oxygen or oxygen radicals in the body (**Figure 2B**). The compounds that result from this reaction have been associated with atherosclerosis and other inflammatory diseases. For example, high concentrations of the reaction product peroxynitrite can result in cytotoxic peroxynitrous acid (Pryor and Squadrito, 1995), hydroxyl radical toxicity (Beckman et al., 1990) and protein fragmentation by nitration of amino acids (Ischiropoulos et al., 1992). Thus, the pro-inflammatory potential of NO generally occurs indirectly and is most prevalent at high concentrations of NO. Peroxynitrite can react directly with some targets (e.g., to activate MMPs) but most of its impacts are through oxidation via a free radical reaction (Szabó et al., 2007). Most of these effects of peroxynitrite promote an inflammatory response. The dual effects at different doses may contribute to the fact that the benefits of NO therapy in adult patients with pulmonary hypertension are still uncertain (Griffiths and Evans, 2005).

The formation of peroxynitrite is expected to be very low in normal, healthy physiological conditions because of the reaction kinetics (Wever et al., 1998). It has been estimated to be in the pico- to nanomolar range. In addition, there are molecules such as glutathione that can protect against oxidation by peroxynitrite (Sies et al., 1997). However, in diseased cardiovascular tissues (e.g., arteries with atherosclerosis), both the oxidative stress and inducible NOS (iNOS) will increase significantly (Wever et al., 1998; Mudau et al., 2012). This will result in higher rates of peroxynitrite production that can potentially cause cell damage. In addition, patients with hypercholesterolemia can have impaired function of glutathione, reducing the protection against reactive species derived from NO (Ma et al., 1997). One question is what impact specific doses of exogenous NO could have if delivered as part of a therapy. Inhaled NO delivered to infants with pulmonary hypertension are typically provided at ≤40 ppm (Weinberger et al., 2001). A clinical trial of inhaled NO (20 and 80 ppm in some patients) showed that the treatment was considered safe as monitored by signs of seizures requiring therapy or intracranial bleeding (Neonatal Inhaled Nitric Oxide Study Group, 1997). Clear toxicity has been observed in the clinic with exogenous doses over 80 ppm. However, modified tyrosine residues, which is a potential concern, were observed in the lungs of infants treated with 20 ppm doses (Hallman et al., 1998; Weinberger et al., 2001). Evidence suggest that there is a dose dependence where inhaled NO at a high 100 ppm dose increased ROSs, but 50 ppm dose exhibited anti-inflammatory properties (Weinberger et al., 2001). Overall, it is clear that the dose must be considered when incorporating NO and NO releasing molecules as part of a vascular tissue engineering strategy.

#### Mechanisms for Endogenous CO

Carbon monoxide is generated naturally in the body when the heme oxygenase (HO) enzyme degrades heme molecules (Tenhunen et al., 1969). CO is most commonly known because high doses, such as those found with smoking, can be pro-inflammatory and higher doses are fatal. This is probably one reason why exploration of biomedical uses of CO started more recently than NO. However, CO in appropriate doses has anti-inflammatory properties (Motterlini, 2007). In addition, animal models with an abnormality in endogenous CO metabolism within cardiovascular tissues contribute to various pathological situations including hypertension, cardiac failure, and inflammation (Wu and Wang, 2005). Further, a patient died because of a unique clinical scenario where HO enzyme generation was reduced, and as a result less CO was present (Yachie et al., 1999). HO-1 has been shown to provide oxidative stress protection by itself (Yachie et al., 1999). However, CO has also independently been shown to provide anti-inflammatory properties. For example, it has been shown that CO acts by inhibiting the expression of the pro-inflammatory cytokines tumor necrosis factor alpha (TNF-α), interleukin-1beta (IL-1β), and macrophage inflammatory protein-1beta (MIP-1β), as well as by increasing the production of the anti-inflammatory cytokine IL-10 (Otterbein et al., 2000). Similar to NO, there are multiple mechanisms of action for CO. The Center for Disease Control (CDC) defines two modes of action which are (a) the hypoxic one that make CO dangerous in high dosage and (b) the non-hypoxic ones from endogenous CO (Wilbur et al., 2012). It is these non-hypoxic mechanisms that are of interest for CO delivery for tissue engineering applications.

Defining specific levels for high and low CO doses, as well as the associated signaling pathways, have been major areas of research. There has been progress, but there are still also many remaining questions. The specific impacts of CO and the associated mechanisms have been reviewed in detail in other review articles and book chapters (Ryter and Otterbein, 2004; Motterlini and Otterbein, 2010; Untereiner et al., 2012; Wilbur et al., 2012; Motterlini and Foresti, 2017). The non-hypoxic impacts range from vasodilatory, anti-apoptotic, and anti-inflammatory impacts important for vascular tissue engineering to neural signaling and prevention of ischemia-reperfusion injury during organ transplant (e.g., kidney). Below, we provide a summary of the some of the important pathways with a focus on those relevant to tissue engineered vascular grafts.

Carbon monoxide binds to fewer targets than NO, but there are still several heme-containing compounds that it has been shown to bind to. These include hemoglobin, myoglobin, soluble guanylate cyclase (sGC), cytochrome c, cytochrome p-450, and NOS. In addition, there are a variety of pathways that have been identified. The toxic effects at high doses have been linked to CO binding to hemoglobin and myoglobin that replaces oxygen (O_2_). CO binds hemoglobin about 220 times stronger than (O_2_), replaces O_2_ from oxyhemoglobin, and forms carboxyhemoglobin (COHb) (Nguyen and Boyer, 2015). As a result, reduced oxygen transport capacity of the red blood cells can inhibit oxygen delivery to tissues. In addition, the binding of CO to myoglobin, which typically provides an oxygen reserve to skeletal muscle cells, has been linked to muscle fatigue (Wilbur et al., 2012). There is the possibility that the negative effects of CO binding to hemoglobin and myoglobin also include eliciting pro-inflammatory effects, such as neutrophil recruitment in the brain and lipid peroxidation (Peng et al., 2013). However, a CDC publication concludes that it is uncertain if plasma inflammation and coagulation markers increase at low to medium levels of CO exposure because some of the literature results appear to be seemingly contradictory. The negative impacts and mechanisms of high CO doses are generally considered well established. However, a Motterlini and Foresti (2017) review article mentions that CO binding to hemoglobin and myoglobin is actually a protective mechanism and that it is the CO that does not bind to these heme compounds and instead binds extensively to cytochrome c oxidase within cell mitochondria that cause the toxic effects. They cite relative CO affinities to the three heme-containing compounds as well as a dog study that suggested that free CO gas is more dangerous than CO bound to hemoglobin. Although the Motterlini hypothesis about hemoglobin toxicity will require further investigation, it suggests the importance of considering biodistribution in tissue and within cells in understanding the difference between toxic and protective CO doses.

**FIGURE 3:**
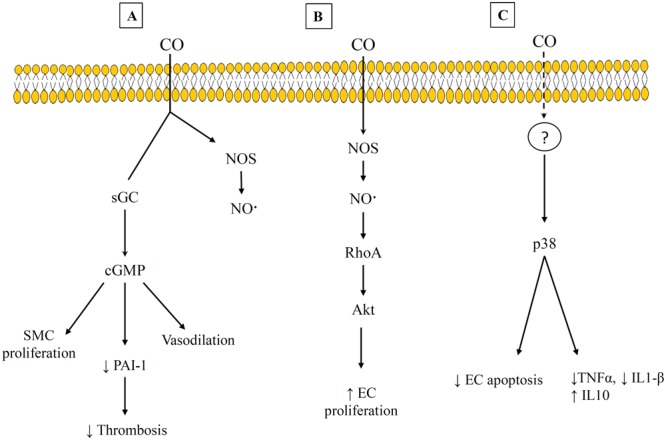
Examples of carbon monoxide (CO) Pathways and heme-binding targets. **(A)** cGMP-dependent pathway that leads to suppression of SMC and decrease thrombosis. **(B)** CO interacts with RhoA and Akt to promote EC proliferation. **(C)** A p38 mitogen activated pathway that exhibits anti-inflammatory effects. References for this figure are listed in the text.

The beneficial effects of CO have been shown to occur through binding to other heme-containing compounds within the cell. Early work focused on CO binding to sGC that leads to increased cGMP production. This is the best characterized pathway, and it has been shown to lead to impacts such as vasodilation, increased thrombolysis, and decreased SMC proliferation (Suematsu et al., 1994; Fujita et al., 2001; Ryter and Otterbein, 2004). However, other heme-containing targets for CO and the corresponding pathways have continued to be identified. Some of these are shown in **Figure 3**. The variety of pathways help to explain the different effects that CO has been shown to have on different cell types. For example, CO appears to promote endothelial cell proliferation but reduce SMC proliferation (**Figures 3A,B**) (Ryter and Otterbein, 2004; Untereiner et al., 2012). Both of these results are beneficial in preventing vessel stenosis and intimal hyperplasia. The suppression of SMC proliferation has been linked to CO binding to either sGC or NOS, and the signaling proceeds through a cGMP-dependent pathway (Morita et al., 1995; Song et al., 2002; Botros and Navar, 2006). This has also been shown to involve transcription factors (e.g., E2F) (Ryter and Otterbein, 2004) and proteins that regulate the cell cycle, including increased levels of the cyclin-dependent kinase inhibitor p21 (Zhou et al., 2005). However, the promotion of endothelial cell proliferation by CO has been linked to CO binding with NOS, and involves Ras homolog gene family member A (RhoA) and Akt (Wegiel et al., 2010). This is one of the pathways that demonstrates an interaction between both the gasotransmitters CO and NO. In addition, CO has been shown to provide anti-apoptotic effects on endothelial cells through a p38 mitogen activated kinase pathway (MAPK) that includes upregulation of anti-apoptotic genes [e.g., inhibitors of apoptosis 2 (IAP2)] (Brouard et al., 2002). Interestingly, CO may also provide dose-specific modulation of cellular apoptosis through mitochondrial swelling. Queiroga et al. (2011) have reported that 10–100 μM CO prevented mitochondrial permeabilization and prevented release of pro-apoptotic factors from the mitochondria but 250–500 μM CO caused mitochondrial swelling.

The anti-inflammatory properties of low CO doses also appears to act through MAPK-dependent pathways. Activation of p38 has been discussed most extensively, but more recently ERK downregulation has also been implicated in these effects (Untereiner et al., 2012). A continuing question about this pathway is how CO initiates the response since it does not bind directly to any of the upstream components. It may be that mitochondria-derived ROS is what initiates the p38 signaling pathway and leads to the anti-inflammatory effects (Otterbein et al., 2000). This is supported by work by Bilban et al. (2006) that showed that macrophages exposed to CO produced temporary bursts of ROS that led to peroxisome proliferator-activated receptor gamma (PPARγ) expression (Bilban et al., 2006). They further showed that blocking PPARγ prevented the anti-inflammatory effects from CO. An anti-inflammatory effect through ROS would be expected to be CO dose dependent since levels of ROS that are too high can lead to a damaging oxidative stress environment.

Carbon monoxide does have a couple of potential advantages for controlling the inflammatory response to vascular grafts despite the increased importance of dose control compared to NO. CO is a more stable molecule than NO (Zhuo et al., 1993). In addition, CO targets only transition metals (e.g., iron) (Motterlini and Otterbein, 2010), unlike NO that interacts with a large number of cell targets. The stability of CO may be a benefit when in the high oxidative stress environment that would typically be found in diseased tissue and after initial graft implantation (Desmard et al., 2012). In this situation, the half-life of released nitric oxide would be very limited and some of the reaction products could cause negative side-effects. Work by Motterlini and Foresti (2017) has suggested that NO is more common in healthy tissues, but HO1-derived CO is more important in tissues with higher levels of oxidative stress where NO is less stable. It is known that NO can increase endogenous CO through an increase in HO-1, and alternatively CO can increase NO generation through binding to NOS (Untereiner et al., 2012). For example, it has been shown that inhaled CO reduced pulmonary hypertension in a mouse model by generating eNOS and NO (Zuckerbraun et al., 2006). Their eNOS deficient control mouse did not exhibit the treatment benefits observed from wildtype mice when CO treatment was provided (Zuckerbraun et al., 2006). These results are supportive of a comparative benefit of CO over NO in an oxidative environment. However, it has also been mentioned that the negative impacts of higher doses of CO (e.g., blood COHb levels ≥2.4%) are more pronounced in patients with previous cardiovascular disease than those who were healthy (Wilbur et al., 2012). The benefits of NO and CO therapy in an inflammatory environment will require more investigation of the dose dependent differences and differences in distribution between particular tissues and cells.

#### Defining High and Low CO Doses

**FIGURE 4:**
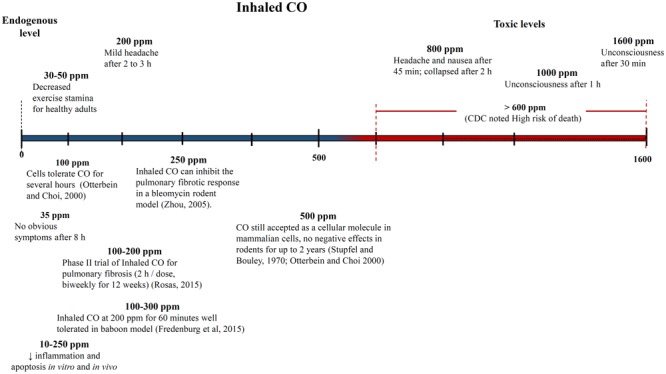
Approximate inhaled CO concentrations (in parts per million) and noted effects. Negative and positive medical effects are shown on the top and bottom, respectively (Stupfel and Bouley, 1970; Zhou et al., 2005; Rosas, 2015). The effects without corresponding citations are from a CDC publication (Wilbur et al., 2012).

**FIGURE 5:**
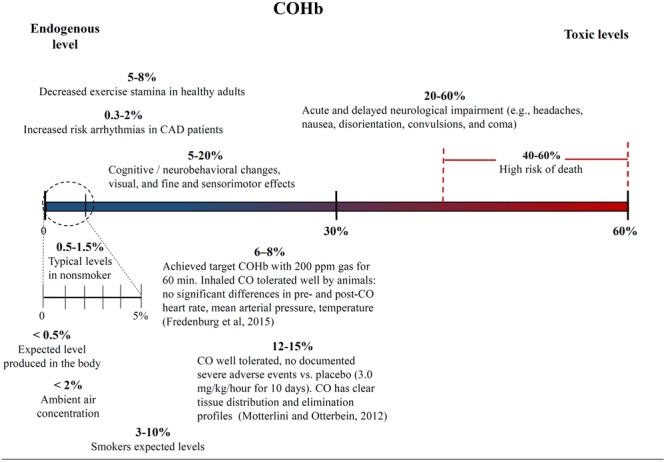
Carboxyhemoglobin (COHb) concentrations in the blood and corresponding medical effects for both environmental conditions and controlled supplementary CO therapy. Negative and positive medical effects are shown on the top and bottom, respectively (Motterlini and Otterbein, 2010; Fredenburgh et al., 2015). The effects without corresponding citations are from a CDC publication (Wilbur et al., 2012).

One of the biggest challenges with interpreting the results of previous CO studies has been determining what constitutes a high and low CO doses. The CO dose has typically been defined as either ppm in the inhaled gas or percent CO bound to a heme compound – COHb *in vivo* in the blood or COMb for *in vitro* tests. **Figures 4, 5** show different studies and reported results at different CO doses. Common endogenous levels are also shown. The endogenous levels range from 0 to 0.5%, but are hard to define. HO-2 is expressed at varying levels in different tissues, with the highest expression in tissues where there are increases in estrogen and glucocorticoids (Wilbur et al., 2012). In addition, HO-1 is induced upon injury, so the specific levels will vary. The higher the HO levels, the higher the amount of CO will be.

While endogenous and high lethal levels can be roughly defined, there are questions about the impacts of CO at levels in between. The chart in **Figure 4** shows that ppm CO in the inhaled gas provides conflicting information about positive and negative levels when different clinical and pre-clinical studies are compared. CO has been reported to demonstrate significant medical benefits at concentrations of the inhaled gas ranging from 10 to 500 ppm (Mann and Motterlini, 2007; Motterlini and Otterbein, 2010), but the CDC report discussed concerns to particular tissues at ppm levels as low as 12 ppm (Wilbur et al., 2012). The results could be influenced by differences in duration and frequency of exposure, so COHb is often used as an alternative measure. **Figure 5** shows COHb levels which are reported less often than ppm CO in the inhaled gas, and are often theoretically predicted instead of experimentally determined (Wilbur et al., 2012). This figure demonstrates that consideration of the COHb levels also exhibits apparent discrepancies for positive and negative levels between studies. For example, the CDC defines 2.4% COHb as the level for the lowest observed adverse effect but other studies have demonstrated beneficial impacts for higher levels of CO [e.g., less than a 15% COHb toxic threshold for a study conducted on healthy, non-smoking, young-men (Benignus et al., 1987)]. It has also been noted that there is significant patient to patient variability in the response to COHb levels, with patients with the same COHb level exhibiting very different responses (Gorman et al., 2003; Grieb et al., 2008). Some patients have been shown to be without symptoms at comparably high COHb levels in their blood as the Benignus et al. (1987) study demonstrated (Motterlini and Foresti, 2017). The difference in levels is important because the projected CDC levels are similar to what a body would be exposed to in normal ambient air, but the higher values in other studies suggests a benefit of additional supplemental CO. Finally, it is even more uncertain how COMb levels found with *in vitro* studies relate to the results in the clinic and in animal models. These studies can provide comparative release rates and biological impacts for different compounds and doses, but an extrapolation to the *in vivo* environment would require mathematical modeling and consideration of mass and fluid transport.

Carbon monoxide biodistribution is likely important to interpret these results and determine optimal levels for supplementary CO doses, but this information is not as well known. This information is also important because different tissues have varying sensitivity to CO. For example, the respiratory track is one of the least sensitive. However, this would stress the importance of local delivery to avoid any systemic effects as well as prevent the binding of most of the CO to hemoglobin in the blood stream so that it can target the heme compounds within the cell (Motterlini and Foresti, 2017).

#### Gasotransmitter Releasing Molecules

Since the dose of NO or CO are very important, the drug delivery strategy is also critical. This is the reason why molecules that degrade to release NO or CO in a controlled manner or in response to a stimuli are being investigated.

Several different NO releasing molecules have been used (Frost et al., 2005). These include diazeniumdiolates and *S*-nitrosothiols (SNO) that release NO when in contact with aqueous solutions such as body fluids. The use of SNOs was likely inspired by the native SNOs found in the body that provide a reservoir of NO (Stamler, 2004). One advantage of SNO is its ability to be regenerated endogenously in the body. Some of these SNOs are compounds that have been synthesized to extend the release time for NO (e.g., by making them more hydrophobic to shield them from metal ion interactions) (Kumari et al., 2014). SNOs have been shown to be active pharmaceutical agents, including inducing vasodilation, and have been incorporated within a wide variety of drug delivery strategies (Liang et al., 2015). These SNOs have also been tested on vascular cells (e.g., SMCs and adventitial fibroblast) including in culture with the antioxidant AA (Gregory et al., 2011). Gregory et al. (2011) found that AA and their particular SNO are complimentary, with AA increasing NO release (Gregory et al., 2011).

A variety of carbon monoxide releasing materials (CORMs) have been developed, as have been described in several review articles (Foresti et al., 2008; Motterlini and Otterbein, 2010). The classes of these CO-releasing molecules can be categorized as aldehydes, boroncarboxylates, metal carbonyl complexes, organometallic compounds, oxalates, and silacarboxylates (Romaõ et al., 2012). The majority of these compounds are metal carbonyls that quickly release CO in the presence of water, but many of which contain heavy metals. There is also a boron carbonate compound that does not contain heavy metals and releases CO at a slower rate. However, CORMs that release CO in response to light are being investigated to allow for a more controlled release. These light responsive CORMs include a manganese complex and organic molecules. CORMs have been shown to elicit anti-inflammatory properties at the right dose for a variety of tissues (Motterlini and Otterbein, 2010). For vascular applications, there are currently limited results but one study showed that a CORM induced extended vasodilation and reduced acute hypertension in explanted rat aortic rings (Motterlini et al., 2002).

#### Incorporation of NO Releasing Materials within Scaffolds

For tissue engineering, incorporation within a scaffold is important and can provide controlled, local delivery of the gasotransmitter (Motterlini et al., 2002; Foresti et al., 2008). NO releasing materials have been incorporated within tissue engineered scaffolds as well as modified PTFE traditional vascular grafts (Gregory et al., 2011). For example, NO has been added to the surface of synthetic scaffolds both covalently for extended delivery (i.e., diethylamine/N_2_O_2_) and non-covalently (spermine/N_2_O_2_) (Frost et al., 2005). The main application of these NO releasing materials incorporated within scaffolds has been as a coating of extracorporeal circulation devices such as those used for hemodialysis (Yang et al., 2015). The primary goal for this application has been to provide a NO-releasing substitute for endothelial cells that can prevent platelet deposition and thrombosis, and the studies have demonstrated success with this goal. For example, Skrzypchak et al. (2007) coated poly(vinyl chloride) tubing used as an arteriovenous shunt in a rabbit model with for diamine-based diazeniumdiolates material that provided clotting protection with DBHD/N_2_O_2_ loadings of 10 – 50 wt%, but not for a lower 2% dose. Toxic nitrosamines can form when many of the NO releasing materials leach out of the coating, but techniques such as lipophilic modification of molecules to reduce leaching and development of new NO releasing materials with lower toxicity concerns are current strategies to overcome this challenge (Yang et al., 2015). Another possible limitation has been that the lack of extended NO release.

Nitric oxide can have other effects relevant for vascular tissue engineering, but these have been less of a focus with the current studies. An NO-releasing poly(diol-co-citrate) elastomer has been shown to reduce SMC proliferation in cell culture and reduce intimal hyperplasia when used a perivascular wrap in a rat model (Serrano et al., 2011). This was performed on a vessel injured by balloon inflation. The incorporation of NO releasing materials within tissue engineering scaffolds that are intended to integrate with the surrounding artery has also been performed. Andukuri et al. (2011) developed a fibrous tubular structure with both endothelial cell binding peptides and polylysine NO donors that reduced platelet adhesion and SMC proliferation but increased endothelial cell proliferation *in vitro*. In a novel technique by Wang et al. (2015) the surface of their TEVG was modified with β-galactosidase to provide the ability to catalyze removal of the galactose group of an NO releasing prodrug (Wang et al., 2015). This technique can allow for greater stability of NO releasing materials injected into circulation. The study grafted the enzyme modified TEVG into a rat aorta and then provided a tail vein injection of the NO prodrug, resulting in increased von Willebrand factor expression from the endothelial cells but also a thicker layer of intimal hyperplasia than the control group without NO. There are also studies that have demonstrated NOS activity from cells seeded within a tissue engineered scaffold. For example, Lim et al. (2008) demonstrate that bone marrow-derived cells pre-seeded on a TEVG differentiated after grafting into the abdominal aorta of dogs and generated eNOS. Another study by McIlhenny et al. (2015) took the extra step of transfecting pre-seeded adipose-derived stem cells with the eNOS gene to decrease the thrombogenicity of their TEVG in a rabbit aorta model. However, most of the TEVG work with NO releasing materials involves *in vitro* studies, and the current TEVG approaches that have shown promise in large animal and clinical trials do not include NO releasing materials (Shojaee and Bashur, 2017). The lack of an established safe therapeutic dose in the body has been described as a current limitation for commercialization of these technologies (Jen et al., 2012). For tissue engineering applications, it will be important to determine how well the TEVGs with NO releasing molecules remodel and integrate in animal models of disease.

#### CORM- Loaded Scaffolds

The incorporation of CORMs within vascular tissue engineering scaffolds has also been limited, and has only involved fibrous scaffolds generated through electrospinning (Bohlender et al., 2014; Michael et al., 2016). CORMs have also been delivered without a scaffold and incorporated within micelles (Peng et al., 2013), but micelles can be washed away. CORMs in scaffolds are the focus of the section below. The local release from the scaffold has the potential to provide controlled delivery to the vascular graft and the surrounding artery and reduce systemic effects. The effective dose can also be reduced because it bypasses the blood stream where the CO would bind to hemoglobin (Romaõ et al., 2012; Heinemann et al., 2014).

It is important that the CORMs can provide controlled release when incorporated within a tissue engineered scaffold. Fibrous scaffolds have several advantages as described previously (Bashur et al., 2013). In addition, these scaffolds can provide a hydrophobic carrier for the CORM materials with proper control of the scaffold composition. This is important for some CORM materials, such as the organic photoactivatable ones that our lab works with that need to avoid hydration to maintain their activity (Michael et al., 2016). The first incorporation of CORMs within a scaffold was performed by Bohlender et al. (2014). Interestingly, their goal with incorporating the CORM was to provide anti-bacterial properties through CO release (Bohlender et al., 2014). The photoactivatable Mn-based material provided the capacity for significant release of CO. In fact, the doses that they provided released CO so quickly that it generated bubbles that were toxic to the fibroblastic cells seeded on them. This is not a surprise since even bubbles composed of air that are generated by ultrasound technology can be toxic to cells (Wu et al., 2008). This study emphasizes the importance of dose control with use of CORMs for a tissue engineering application.

The first incorporation of CORMs within a scaffold for tissue engineering was performed by Michael et al. (2016). The CORM was blended with a hydrophobic polymer and electrospun to generate a fibrous mesh. Importantly, this mesh allowed a time frame in which vascular cells can be seeded prior to activation and then the construct was used for a tissue engineered vascular graft application. The time frame with the specific CORM tested in this study was only 1 h, and the strategy may need to be modified to provide a longer time frame. However, it is important that the cytotoxic effects noted in the Bohlender study were not observed with the lower CO doses and slower CO release that was used in the study by Michael et al. (2016). They did not find a significant decrease in SMC viability with 2% (w/w) CORM incorporation and activation. The SMCs were also shown to attach and express phenotypic markers. Currently, there is limited research on the impact of CORMs and CO dose that would explain the results of these studies. However, Nobre et al. (2016) evaluated the ability of CORMs to deliver CO to bacterial and mammalian cells and investigated the toxicity to mammalian cell for several CORM compounds. They found that most CORMs and the released CO were non-toxic to eukaryotic cells even at high concentrations that resulted in bactericidal activity. Specifically, the antimicrobial effects of the metal-based CORM (CORM-3) were noted at 50-fold lower concentrations than those that were toxic for eukaryotic cells (Desmard et al., 2009). This interesting finding suggests that CO may be able to simultaneously provide antibacterial properties as well as support cell function in vascular grafts at appropriate doses, although additional research is required. Finally, the impact of the inflammatory environment in the body on the tissue engineering use of CORMs has not yet been determined.

#### Outlook for Gasotransmitters

Gasotransmitters have shown promise for improving TEVGs and represent the most common approach to deliver pharmaceutical compounds with tissue engineered vascular grafts. However, these studies are in the early stages. One of the critical areas of research will be verifying the appropriate doses and potential side effects. Careful control of the dose and investigation of the pharmacodynamics are especially important for NO and CO delivering compounds because of their concentration dependent effects. This will include an assessment of the biocompatibility of the NO donor or CORM itself in case it leaches out of the scaffold, the released gas, and the degradation product after activation and release of the gasotransmitter. This is demonstrated by Nobre et al. (2016) where they showed that there was no correlation between the bactericidal activity and the ability of CORMs they tested to release CO into the medium. New CORMs have been developed that provide lower toxicity potential. As an example, the CORM used by Michael et al. (2016) is an α-diketones that has been shown to have limited cytotoxicity for KG1 myeloblast cells and rat SMCs within doses up to 40 μM tested in cell culture (Peng et al., 2013). The desired rate of CO release still requires further investigation by the CORM field. Finally, the degradation product of the Michael et al. (2016) CORM is anthracene, which has a low toxicity for aromatic compounds. However, detailed analysis of the toxicity profile will be need for any new anthracene derivatives or other CORM molecules that will be employed. Overall, more work is needed, but these delivery strategies may allow for generation of a viable tissue engineered vascular graft that would provide needed options for patients with coronary or peripheral artery disease.

## Bioabsorbable Stents

Tissue engineering and drug delivery principles can also be applied to stent grafts, which are another treatment option for small-diameter vessels. The clinical use of stents has continued to increase due to the less invasive nature the endovascular surgery required for stent placement. However, stents still have limitations. For the peripheral arteries in the leg, a significant concern is stent fracture or movement due to the force applied by the surrounding muscle (Sarkadi et al., 2015). This is not a major concern for the coronary arteries. However, restenosis is a concern for stent-grafts applied to both types of small-diameter vessels (Ormiston and Serruys, 2009).

The simplest type of stent is bare metal. There are several bare metal stents on the market, but these exhibit high restenosis rate from 15 to 60% within 12–24 months (Chen et al., 2006; Schillinger et al., 2007). For this reason, drug-eluting stents are commonly used. Most drug-eluting stents have a polymer coating that releases the anti-inflammatory drugs sirolimus or paclitaxel. Metadata analyses have demonstrated an advantage of sirolimus and paclitaxel eluting stents compared to bare metal stents (Biondi-Zoccai et al., 2009). However, the SIROCCO trial found no significant difference in 2-year restenosis rate between sirolimus-eluting stents and bare metal ones, with >20% restenosis rate for the drug-eluting stent (Duda et al., 2006). Overall, the long-term outcomes for drug eluting stents will require further investigation, and could be improved (Antoniou et al., 2014).

Bioabsorbable stents are currently being investigated as an alternative to drug-eluting stents, with the goal of reducing the rate of restenosis. One consideration with the anti-inflammatory drugs in drug-eluting stents is that they are typically released over 30 days, and exhibit an initial burst release (Tzafriri et al., 2012). However, it is not clear how long-lasting the effects of these drugs in the diseased artery are after the drug is no longer available. The biodegradable polymer coating on the stent can be adjusted to extend the controlled release, including up to 90 days with the CYPHER^®^stent (Abizaid and Costa, 2010). However, this is still a relatively short release compared to the life of the graft. In addition, the polymer coating will dissolve over time exposing the metal struts. The increased narrowing of the vessel lumen at extended times post-stenting (e.g., >7 years) has been linked to continued inflammation from cell contact with the metal stent (Ormiston and Serruys, 2009). Bioabsorbable stents, however, rely on tissue engineering principles. The goal is have the stent degrade over time while generating a functional endothelium, and resulting in integration with the native tissue.

Bioabsorbable stents have been constructed entirely out of a degradable polymer as well as out of a resorbable metal (Ormiston and Serruys, 2009). The first bioabsorbable stent used in clinical trials consisted of poly (L-lactic acid) (PLA) without any anti-inflammatory drugs. There is also current work investigating a PLLA-based graft that also elutes paclitaxel (Lan et al., 2014). There are two companies that sell bioabsorbable stents, Biotronik and Abbott. The Biotronik stent uses a magnesium alloy that degrades over 12 months along with a sirolimus-eluting coating (Haude et al., 2016). The Abbott stent is a PLA-based stent with a drug eluting everolimus coating. These bioabsorbable stents have shown promise and received FDA approval, but there are results that indicate that some aspects of these stents can still be further improved (e.g., thrombosis levels) (Stone et al., 2016). They are in the early stages of development and will continue to be further investigated. Bioabsorbable stents are further demonstration of the use of tissue engineering and drug delivery principles for treating patients with diseases of their small-diameter arteries.

## Conclusion

In summary, this article discussed the clinical need for vascular tissue engineering, the intersection of drug delivery with vascular tissue engineering, and how tissue engineering and drug delivery concepts are also being applied to stent grafts. With tissue engineering, one of the main goals is controlling inflammation to allow initial generation of tissue, instead of preventing inflammation. Different pharmacological agents and scaffold delivery approaches have been used for vascular tissue engineering. These include the anti-oxidant compounds and gasotransmitters reviewed in this article. The anti-inflammatory compounds in particular, are often presented for an extended period of time as part of the scaffold, instead of a traditional drug delivery approach. However, there are comparably few pharmacological compounds that have been investigated for vascular tissue engineering. The steps in the inflammatory process offer potential targets for future investigation. Overall, these pharmacological strategies represent the potential to promote the formation of a functional endothelium and maintain graft patency. As tissue engineered vascular grafts and biodegradable stents are further pursued, they will hopefully extend the life-time of vascular surgical replacements.

## Author Contributions

KW and CB contributed to the initial draft of the paper and revisions. KW prepared the figures and tables.

## Conflict of Interest Statement

The authors declare that the research was conducted in the absence of any commercial or financial relationships that could be construed as a potential conflict of interest.
